# Sympathetic Ophthalmia

**Published:** 1874-05

**Authors:** A. W. Calhoun

**Affiliations:** Atlanta, Ga.


ATLANTA

MEDICAL AND SURGICAL JOURNAL.

VOL. XII.] MAY—1874. [No. 2.

### Original Communications.

### SYMPATHETIC OPHTHALMIA.

*Read before the State Medical Association of Georgia, at Thomasville, April 1st*, 1874.

BY A. W. CALHOUN, M.D., ATLANTA, GA.

In the whole range of diseases of the eye, there is none regarded with more just fear than that condition of things known as Sympathetic Irido-Cyclitis—in other words, a peculiar affection transported by means of the ciliary nerves from the one, the injured or diseased organ, to the other or hitherto sound organ. As to the truth and force of this remark, each and all of you will doubtless bear witness, for but few physicians, having a reasonable professional career, have failed to have brought under their supervision, one or more such cases, well illustrating the fact that no disease is so sure in its dread results, when once the flame has been fanned into existence.

Since such melancholy consequences are known to take place, and always anticipated under certain circumstances, it would be a rational supposition that the profession should be ever ready to combat the disease, and strip it of at least some of its attendant evils, and yet how often do we come in contact with unfortunate individuals whose vision has been irretrievably lost by such an inflammation.

As a rule, sympathetic inflammation in the one eye, is caused by an *injury* inflicted upon the other, the injury preceding the attack of the inflammation by an interval ranging from a few months to many years. Chiefly, wounds that tear and penetrate the ball, foreign bodies, as pieces of wood, stone, iron, etc., also a heavy blow directly upon the eye itself, are the causes of the destruction of the first diseased eye. So often is it the case that this particular inflammation in the one eye is the result of a traumatic injury in the other, that we almost involuntarily conclude this to be the fact, whenever such an affection presents itself to our consideration. The most dangerous wounds are those situated at the junction of the cornea and sclera, passing through and striking the ciliary processes; particularly dangerous, when at the same time, a prolapse of the iris has taken place, the prolapsed portion becoming fastened in the wound, producing a constant tension upon the iris.

Græfe and Critchett report cases where the inflammation was the result of an operation for the extraction of cataract, but seldom, however, are we authorized to attribute the disease to the influence of an operation.

Generally, the first appearance of the disease in the second eye, occurs after the injured one has been very seriously affected or completely destroyed, vision having been in a large majority of cases, entirely lost.

The patient at first observes that his sight is becoming imperfect, which continues for a variable period, when the signs of iritis come on, such as ciliary injection, slight, diffused opacity of the cornea, a cloudiness of the aqueous humour, discoloration of the iris and adhesions between its posterior surface and the lens. Usually the entire posterior surface of the iris becomes adherent, and where this is not the case, the collection of the secretions behind the iris bulges its periphery forward, with the center sunken, by reason of the pupillary margin being held down to the surface of the lens—the pupil becomes smaller and exudations fill up the entire space producing occlusion, the anterior chamber diminishes in size; in short, all the symptoms of irido cyclitis develop themselves.

The cause of vision becoming unsettled in the very beginning of the inflammation is that the vitreous humour becomes muddy and unclear, which increases as the disease progresses, preventing a satisfactory view with the ophthalmoscope even before any positive external signs are apparent. Later in the course of the inflammation, the lens itself assumes an opaque appearance.

The tension of the eye increases slightly in the beginning, (according to some observers,) then follows a decided and continually increasing softness of the ball until finally, in the larger number of instances, phthisis bulbi ensues.

The subjective symptoms, pain, photophobia, etc., are in many cases quite severe. More frequently, however, these do not come very prominently forward.

The progress of the disease is always slow, and sometimes when the inflammation has ceased, a certain amount of vision is still left, but this is unusual, the ball growing softer and softer until it is entirely destroyed. McKenzie relates cases where the primarily diseased eye possessed tolerable vision, while the sympathetically diseased one was totally bereft of it.

Most frequently, an interval of from four to eight weeks is passed over, between the primary injury and the appearance of the sympathetic inflammation in the second eye. This is however, the earliest moment at which it has been known to take place. By no means is this the latest period. Not unfrequent is it, that many years intervene between the two. In a case where I extirpated the eye-ball a few days since, the first symptom of sympathetic inflammation came on in the second eye four years ago, twenty years after the first had been destroyed by being shot with an arrow. In another, the injury was received when four years of age by a cut from a knife-blade, and just now, after a lapse of thirty-five years, (patient being thirty-nine years old) is the sympathetic inflammation being ushered in. In fact, so long as the deep-seated chronic inflammation in the affected ball exists, having at the same time as a constant symptom, pain upon slight pressure over the ciliary bodies, just so long is the secondary or sympathetic trouble to be apprehended. The traumatic inflammation may have completely subsided and the injured eye may have shrunken away (phthisis bulbi) and years of absolute exemption from whatever unpleasant sensation may have been enjoyed, when suddenly, without apparent cause, fresh inflammation is set up in the first or injured eye, threatening to extend rapidly to the other, bringing about a sympathetic affection. Particularly is this to be feared, if a foreign body has remained within the eye, or the choroid coat has become ossified, the lens or vitreous calcinated.

I have now a case, where, during the war (in 1863) a minnie ball striking the right side of the face, passed through the orbit behind the globe, injuring the latter in its passage, (it was supposed that the optic nerve was severed) and lodging and remaining imbedded somewhere in the bones entering into the formation of the orbit. Vision was at once thoroughly lost and the eye gradually shrank away to a mere stump. But the coats of this stump have become hardened, ossified, and it is now a constant source of irritation, now and then forcing the uninjured eye to share a portion of the irritation, to relieve which extirpation will sooner or later become necessary.

It was for a long time supposed that sympathetic inflammation or irido-cyclitis was transported from the diseased eye to the other by means of the optic nerve, and this would look reasonable, since a nervous connection exists between the two eyes through the two nerves. But the investigations of Arlt, H. Mueller, Bowman and others, have clearly proven that the ciliary nerves are the tracts by and over which the disease is borne from one to the other. The fact, that in many cases the sympathetic inflammation first breaks out at a point upon the ball, exactly symmetrical with the place of injury upon the first diseased eye, is another argument in favor of the belief that it is transmitted by means of the ciliary nerves. If, upon extirpating the bulbus in the case of the man who lost his eye during the war, by being shot with a minnie ball, the optic nerve is found severed, as was decided by the several surgeons who had him in charge, it will be a decisive argument that it is not through the optic nerve that the inflammation in the second eye is induced.

It is only within a comparatively few years that sympathetic inflammation has been clearly understood, and no doubt now exists, that many of the cases of reported cure of this affection by the various modes of treatment that have from time to time been brought before the profession, were not, in reality, cases of sympathetic irido-cyclitis, but some other affection, which yielded partially or completely to the treatment brought to bear upon it. *Nothing*, short of excision of the diseased eye-ball, is a preventive of sympathetic disease in the other.

Critchett, of London, first proposed, and it is now quite extensively practiced, that the eye, affected to the extent of bringing about its destruction, whether diseased by injury or otherwise, should be extirpated *even before any* appearance of inflammation has manifested itself in the other. *Unquestionably* is this mode of treatment indicated, when, after an injury, the eye is lost under the symptoms of irido-cyclitis, the ball becoming soft, and the slightest touch in the region of the ciliary processes producing pain. In such a condition, the one eye should be removed at once, even before the very slightest symptom of an inflammation has arisen in the second. By such a procedure, it is true, many a *diseased* eye *might* be unnecessarily sacrificed, for we not unfrequently find all the tangible conditions in the one eye for the production of sympathetic inflammation in the other, and yet it fails to take place. Still the sacrifice of a diseased and already blinded eye, which must sooner or later, under the process known as phthisis bulbi, necessarily shrink away and leave behind a most unsightly disfigurement, weighs but little when compared with the risk and danger of complete blindness, a consequence of sympathetic disease (irido-cyclitis) taking place in the previously sound eye.

On the other hand, care should be taken, not to be *too free* in the enucleation or extirpation of an eye-ball, unless *some* of the evidences of deep-seated inflammation are or have been present, for the moral impression made upon the stoutest individual by the extirpation even of a blinded eye, is very marked. And then again, an artificial eye falls far short of supplying every wish, although the cosmetic effect may be in many instances almost perfect.

But another and important question arises, viz: If, when the patient comes under treatment, sympathetic inflammation has already been more or less fully established in the second eye, should the first or diseased one be removed with the view of checking the inflammatory process? It was once thought that even then the extirpation would check the advance of the inflammation, but this idea has long since been given up. Yet, it would be a great temptation to resort to such an operation, when we know the cause of the secondary inflammation to lie in the first diseased organ, and that there might be a possibility, however bare, of lessening the one by the removal of the other. If a tolerable amount of vision existed in the first diseased or injured eye, (as is sometimes the case,) then it would be worse than folly to destroy this by the extirpation of the eye, with the prospect of the other being completely blinded by sympathetic inflammation going on within it, and a doubt also existing as to whether the operation would be of service or not.

Little is also to be expected from an operation upon the sympathetically inflamed eye, although an iridectomy has for a long time been thought to be an excellent remedy against such an affection.

Experience has taught that after the disease has appeared in the second eye, the best plan is simply to wait till the inflammation has entirely subsided before resorting to any operation, since any surgical interference earlier than this, results in no good. The extirpation of the first diseased eye can at any time be made, with the hope of affording assistance to the second, provided, the acute inflammation is not too severe, or the amount of vision in this eye does not contra indicate such a procedure.

Even after every symptom of inflamation has disappeared from the sympathetically inflamed eye, the iridectomy does but little good on account of the atrophic condition of the iris, tearing when seized by the forceps. Under such circumstances, Graefe recommends, at the same time an iridectomy is made, to extract the lens, since it is either opaque or the pupil is filled up with an exudative membrane. This gives oftentimes much trouble, for it is not only difficult to cut out a portion of the iris, but a considerable amount of the cortical substance of the lens remains behind, a good deal of which can however be removed by carefully rubbing the cornea, under the closed lid, or by the introduction of a spoon suited to the purpose.

In many cases, only a painful irritation is present. The light gives the eye slight pain, the flow of tears is increased and the eye is reddened by the least amount of exercise of the organ. The patient is unable to hold out any length of time at work, by reason of the slight pain coming on, and the vision for the moment failing, in consequence of the muscle of accommodation (the ciliary muscle) giving away. These symptoms may continue for a long time without passing over into an inflammatory stage, and it is often difficult to decide whether it is sympathetic or not. But if there is cause to fear such an inflammation, the extirpation of the injured ball would be nothing more than following the dictates of prudence. And if by the removal of the first diseased eye, these symptoms, or even more outspoken ones, rapidly disappeared, it would be a striking proof of the sympathetic character of the affection, and that the diseased eye was the cause of it.

Sometimes, after the extirpation of the first diseased eye, the second or sympathetically inflamed one recovers very slowly, a certain amount of irritation still remaining.

In such cases, it is the favorite practice of Arlt, in Vienna, to put the patients upon what he terms "*the dark cure,*" ("*Dunkel Cur,*") which consists in keeping the patient confined to a moderately darkened room for some weeks after the operation, until every symptom of irritation has passed away. By such means the eye is protected from the influence of the light, and is kept at absolute rest for a sufficient time—a thing that should be enjoined upon every patient. At the same time, according to discretion, he should be put upon a mercurial course.

Six weeks or two months should be allowed to intervene, after extirpating an eye, before wearing an artificial one. It may be well in conclusion, to note that a few cases are reported, where the wearing of an artificial eye was the cause of sympathetic inflammation. More liable to this are those cases where, from injury, the eye has collapsed, (Phthisis Bulbi,) and the artificial eye has been worn upon the stump. This results from the fact that the glass comes in contact with the portion of cornea that may be remaining, which, being in direct connection with the ciliary nerves, is irritated or inflamed, and the irritation is at once transmitted to the other eye. In all the reported cases of sympathetic inflammation arising from this cause, the symptoms were at once relieved by the removal of the artificial eye.

